# Design and Experimental Setup of a Robotic Medical Instrument for Brachytherapy in Non-Resectable Liver Tumors

**DOI:** 10.3390/cancers14235841

**Published:** 2022-11-26

**Authors:** Paul Tucan, Calin Vaida, Daniel Horvath, Andrei Caprariu, Alin Burz, Bogdan Gherman, Stefan Iakab, Doina Pisla

**Affiliations:** CESTER, Technical University of Cluj-Napoca, 400114 Cluj-Napoca, Romania

**Keywords:** robotic brachytherapy, accuracy, device maintenance, ex vivo liver experiments

## Abstract

**Simple Summary:**

This paper presents an experimental study on a brachytherapy multi-needle insertion device, manipulated using a collaborative robot, to assess a set of characteristics: accuracy, needle deflection due to resistance forces, instrument and needle wear and the influence of insertion speed and rotation during needle manipulation inside the liver parenchyma.

**Abstract:**

This paper presents a study regarding the design and the experimental setup of a medical robotic system for brachytherapy using tribology analysis. The robotic system is composed of a collaborative robotic arm and a multi-needle brachytherapy instrument controlled using a unified control system embedding a haptic device and force-feedback. This work is oriented towards identifying the technical characteristics of the system components to determine the accuracy of the procedure, as well as using different scenarios for needle insertion in ex vivo porcine liver tissue in order to determine the forces required for insertion and extraction of the needle and the friction coefficient that accompanies the previously mentioned forces. Subsequent to the computation of the friction forces, the normal forces and the wear during the needle insertion are determined with the scope of predicting the lifecycle of some components of the medical device.

## 1. Introduction

Statistics published by the International Agency for Research on Cancer (IARC) state that, worldwide, one in five people will develop some form of cancer during their lifetime [1]. Additionally, the World Health Organization also reported that cancer accounts for “nearly one in six deaths” worldwide [2]. According to Dalmartello et al. [3], cancer mortality rates have been on the decline over the past three decades, but the disease continues to be among the leading causes of death (together with ischemic heart disease, Alzheimer’s disease and other dementias, stroke, etc.). The American Cancer Society [4] estimated at the beginning of 2022 that over 1.9 million new cases of different types of cancer will be reported for 2022 and over 0.6 million deaths in the United States alone. Siegel et al. [5] provides a numeric estimation of newly developed cancers in 2022, estimating 395,600 new cases of genital system cancer (highest scoring prostate cancer), 343,040 new cases for the digestive system (highest scoring colon, pancreas, rectum and liver), 290,560 new cases of breast cancer, and 254,850 new cases for the respiratory system (highest scoring lung and bronchus).

Hepatocellular carcinoma (HCC) is the most common primary malignancy of the liver, with a high incidence rate in both sexes and among the highest lethality index, 0.93/1. The curative treatment options for HCC are surgical tumor resection (which can be applied in approximately 20% of the total number of cases) and liver transplant (which is restricted to excellence medical centers). For the remaining 80% of cases, there exist multiple combined therapeutic strategies from loco-regional treatments (transarterial chemoembolization (TACE), radiofrequency ablation (RRA), brachytherapy) to systemic ones, but due to the complexity of the disease and the plethora of individual specific conditions, there is no consensus on the best therapeutic treatment [6,7].

Recent studies in HCC interventional radiotherapy reveal that image-guided brachytherapy can overcome the limitations of TACE and RFA including location and size of the targeted tumor [8].

Brachytherapy uses a series of specially designed needles to deliver the radiation inside the patient directly in the targeted tumor [9,10]. Brachytherapy is usually performed manually by a surgeon or oncologist, by using previous CT (Computer Tomography)/MRI (Magnetic Resonance Imaging) scans, and is able to insert brachytherapy needles inside the tumor using real-time ultrasound guidance. The success rate of the procedure is often dependent on the skills of the surgeon/oncologist, requiring high accuracy and spatial vision from the medic.

Even though the placement accuracy of the needle during the brachytherapy is critical for the success of the treatment, there are other aspects that need to be carefully analyzed such as needle deflection during the insertion, shifting of the tissue during the insertion and the requirement to insert multiple needles to improve the effectiveness of the treatment [11].

To improve the success rate of the brachytherapy procedure, robotic systems were developed for accurately guiding and inserting the needles supervised by powerful imaging tools used to recreate the operational workspace and place the radioactive seed as close to the tumor to obtain the best results and at the same time to monitor the needle insertion in real time to overcome the deflection when passing through non-homogenous tissues.

Dai et al. [12] divides the brachytherapy robotic system into four categories with respect to the imaging method used: ultrasound (US)-guided systems [13,14,15,16], MRI-guided systems [17,18,19], CT-guided systems [20,21,22,23] and additional image-guided systems [24,25,26,27]. Lin et al. [28] proposes a multi-DoF (Degrees of Freedom) robotic system for liver brachytherapy. The medical device consists of three mechanisms: a gantry-type mechanism for positioning, a two DoF orienting mechanism and a needle actuator equipped with an axial force sensor, a real-time US probe and a brachytherapy module. During the laboratory experimental tests, the robotic system proved a mean positioning error of 0.69 mm, with a standard deviation of 0.33 mm. Pisla et al. [29] proposes a parallel robotic system able to guide a multi-needle instrument and a US probe, the system uses two parallel modules of five DoF to guide the insertion instrument and the US probe. The needle-inserting instrument and the one guiding the US probe have four DoF. Pei et al. [30] proposes a smart needle system comprising a hollow canula actuated by two servomotors. The system was built with the scope of minimizing the needle deflection and improving the flexibility of seed placing. The authors state that the smart needle system has no influence on the dosimetry and, during the experimental tests, it performed with high accuracy, reliability and versatility in different medical tasks. Varnamkhasti and Konh [31] propose a flexible 3D printed percutaneous needle with embedded motors. The needle is controlled using a mobile control system. The system is able to reach the target point while avoiding obstacles by controlling its angular deflection and axial motion.

This paper proposes a new robotic system for brachytherapy consisting of a commercial collaborative robotic arm and a novel multi-needle insertion instrument. The robotic arm and the insertion instrument are controlled using a unified control system able to provide force-feedback using a three-axis force/torque sensor and a haptic device. The robotic system can accurately insert up to six brachytherapy needles during a single loading and using the force/torque sensor may correct the needle orientation during the insertion in order to reduce the deflection of the needle.

## 2. Materials and Methods

In a strategic document published in 2022 [3], the five-year survival rates for different cancers were listed (see Table 1), emphasizing two important aspects:

(1) —The overall survival rate is not an average calculation but rather an indicator of detection efficiency, pointing out that some cancers are “silent” ones, being detected only in later stages when the treatment options are limited;
(2) —The incidence points out the potential spread and evolution in numbers.

Interventional radiotherapy is a viable treatment solution, especially for those tumors located deeply into the patient’s body, such as the retroperitoneal area or the mediastinum, where access to the tumor area imposes a highly invasive surgery that cannot be tolerated by most cancer patients. New generations of robotic devices have the technical capabilities required by the procedure, their integration as therapeutic options having the potential to improve survival rates in the next decade. To emphasize the potential therapeutic benefits of robotic brachytherapy and to motivate our research, a set of clinical cases from the Oncology Institute “Ion Chiricuta” Cluj-Napoca are listed in Table 2, extracted from patient files [32].

A short overview of the robotic system for brachytherapy proposed is presented followed by a short presentation of the medical protocol for the brachytherapy, a theoretical study regarding the accuracy of the procedure, a systematic analysis of the main robotic system characteristics and experimental tests using ex vivo tissue.

### 2.1. Robotic System for Brachytherapy

**Figure 1:**
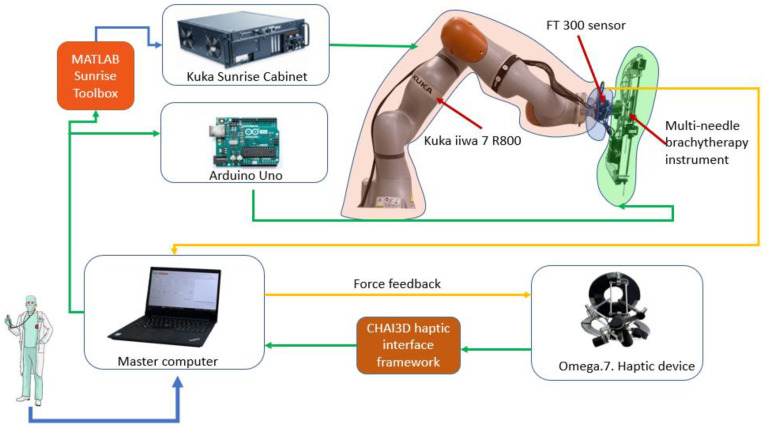
The robotic system architecture.

Figure 1 presents the architecture of the robotic system for brachytherapy. The medical doctor performs the brachytherapy procedure using the master computer which controls the robot (Slave). The computer receives data from the external peripherals (Omega.7 using CHAI3D (Release 3.2.0) [33] and FT 300 (Robotiq, Bron, France) [34] using a designed driver) and communicates with the Kuka Sunrise Cabinet (Kuka Ag, Cluj-Napoca, Romania) using MATLAB Sunrise Toolbox [35] that controls the Kuka iiwa 7 R800 arm (Kuka Ag, Cluj-Napoca, Romania) [36] and with the Arduino (Arduino, Cluj-Napoca, Romania) [37] microcontroller that controls the motors of the brachytherapy instrument [23,38,39,40].

The robotic arm provided by Kuka was chosen after a critical analysis of the existing commercial solutions. Some of the technical aspects required for a robotic system to be used in the brachytherapy are described below:

- Payload—the total mass of the brachytherapy instrument is 1.8 kg;
- Repeatability—in order to achieve high accuracy, a high repeatability was required;
- Light weight—the robotic arm is mounted on a table near the CT, and it also needs to be easily removed from the operating workspace;
- Collaborative—in order to be able to use the robot inside the human workspace, the system must have Human Robot Interactivity (HRI).

Kuka LBR iiwa 7 R800 has a payload of 7 kg, ±0.1 mm repeatability, it is a lightweight robot with a mass of approximately 24 kg, and it contains HRI characteristics required by the working conditions. Apart from the characteristics defined above, the system must be controlled in real time using a MATLAB toolbox, and most essentially the robotic arm also has a medical version (Kuka LBR Med) that can easily be integrated in the medical environments, reducing some time-consuming activities required to obtain permissions to use the robotic system in the medical environment.

The master computer contains a graphical user interface, designed using MATLAB [41] that unifies the control system for the Kuka LBR iiwa robot and the one for the brachytherapy instrument. In order to send real-time data, the Kuka Sunrise Cabinet, which is only programmable offline, and MATALB Server Toolbox were used to create a communication protocol between an application running on the Kuka controller and a server created on the master computer, able to send coordinates and receive position and torque data from the Kuka controller.

**Figure 2:**
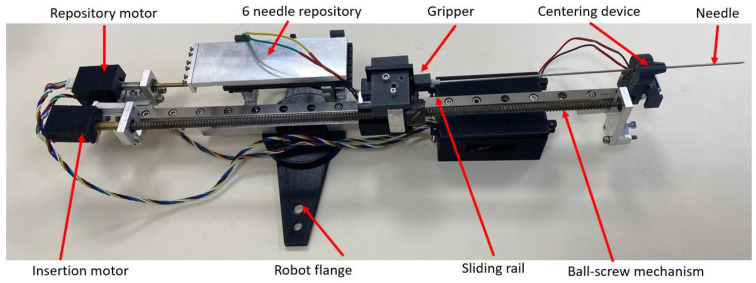
Multi-needle brachytherapy instrument.

**Figure 3:**
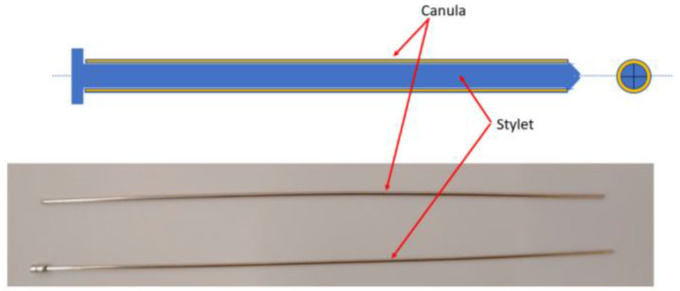
Components of the brachytherapy needle.

Figure 2 presents the multi-needle instrument for brachytherapy. The instrument is capable of inserting up to 6 needles during a single loading. The instrument uses two DC motors to select the needle from a sliding repository containing 6 needles and to insert the selected needle. When loading a needle, the repository slides towards the gripping mechanism which employs a solenoid to open the upper jaw of the gripper and constrain the needle between the jaws. A ball-screw mechanism with a fine pitch (0.8 mm) is used to insert the needle from insertion point to target point through a centering mechanism also actuated by a solenoid; the centering device is used to create a fulcrum point for the needle during the insertion and also to keep the cannula still while the insertion mechanism retracts the stylet of the needle. A closer look at the needle and its components is presented in Figure 3, while the working principle of the entire robotic system may be seen in the next section.

### 2.2. The Medical Protocol for Robotic Brachytherapy

A medical protocol for the robotic brachytherapy was developed together with medical experts. It consists of 20 steps divided between the three main treatment stages: (1)—preplanning; (2)—treatment; (3)—follow-up and monitoring (Table 3).

The medical protocol is designed for minimal human intervention. While all the steps are carried out using automated algorithms (to eliminate human error), the medical experts will acknowledge their correct completion to enable the system to perform the next operation. Thus, the role of the medical expert is changed towards a supervisory role, giving him/her full control of the procedure, which is enhanced by the accuracy of the robot (greater than the manual one) and the possibility of using real-time CT scanning (which cannot be performed in the manual approach).

**Figure 4:**
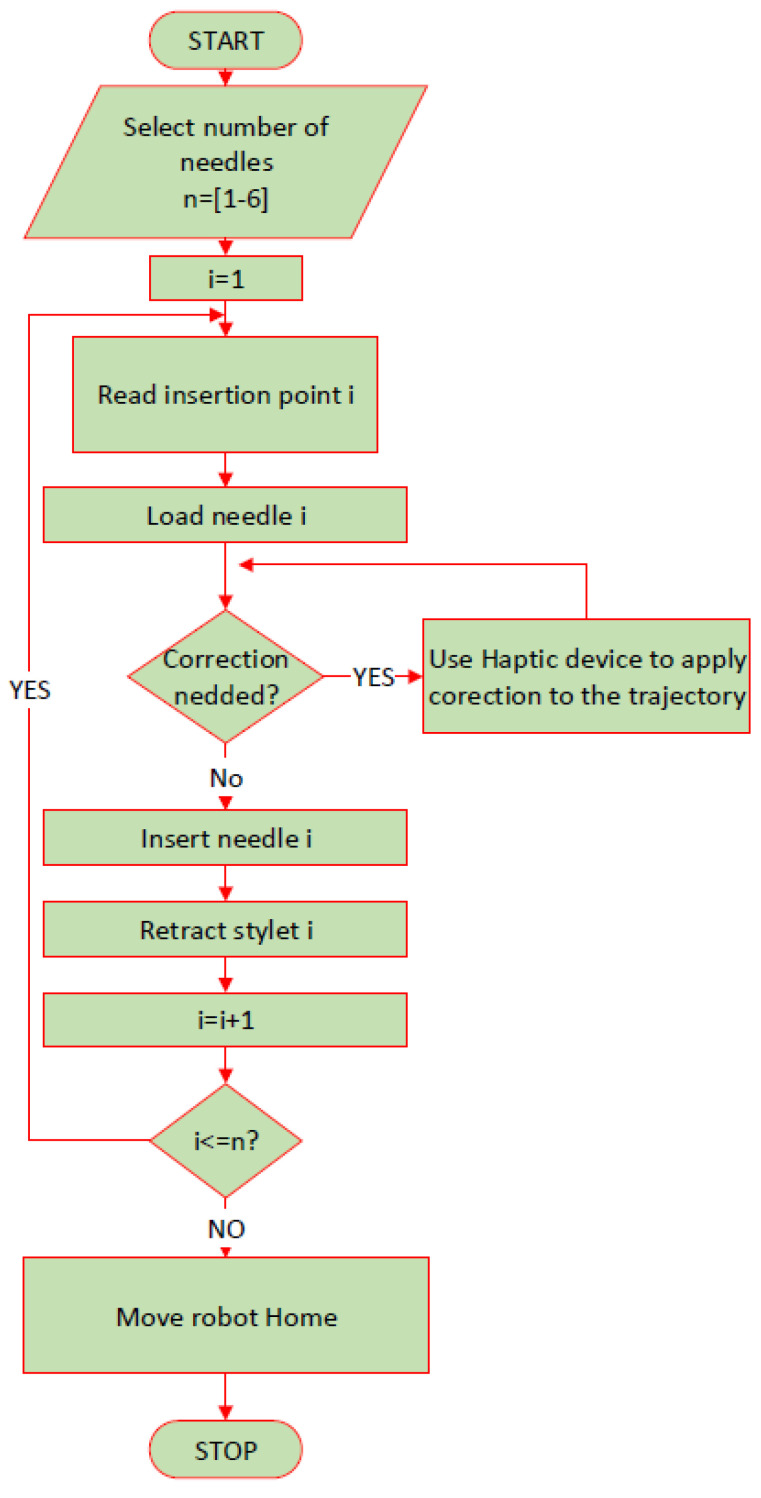
Robotic brachytherapy protocol.

The personalized protocol for the robotic brachytherapy is detailed in Figure 4. The number of the needles required for the procedure is given as an input by the operator and the needle number *i* = 1 is selected. Using a mobile memory unit (USB), the coordinates of the point selected by the surgeon as insertion and target points are transferred from the CT to the user interface of the robotic system. The interface reads the insertion point and the target point for the *i* needle and computes the required orientation for the needle to reach the target point passing through the insertion point on a linear trajectory. After trajectory computation, the robot places the tip of the needle *i* in the insertion point and waits for the operator to confirm the correctness of the positioning, and if corrections are required, then the operator may use the haptic device to reposition the needle until the optimal insertion point is achieved. Afterwards, the instrument inserts the needle “*i*” towards the target point. The operator may set the inserting depth for which the system will require an additional CT scan for needle path validation. If the deflection of the needle is too high, the needle is retracted, and the trajectory is corrected. If the needle is on the desired path, then the insertion continues until the needle reaches the target point. Upon reaching the target point, the robotic system will wait for operator validation. If the optimal target point is reached, then the instrument retracts the stylet of the needle, the operator removes the stylet from the gripper of the instrument and the next needle is loaded until the number of needles selected at the beginning of the procedure is reached. If during the procedure, an emergency requires the robotic system to be immediately removed from the operating workspace, the emergency button of the system is pressed, and the robotic system enters the hand-guiding mode, and it can be easily removed from the operating area.

### 2.3. Systematic Analysis of the Robotic System Characteristics

Starting from the medical protocol and the accuracy of the robotic system, a set of requirements were defined and subsequently analyzed using the AHP (Analytical Hierarchy Prioritization) method to establish their individual criticality level [42].

Monitored accuracy. As illustrated in Section 2.1, the accuracy for the needle positioning is critical and must be monitored externally. As the system is designed to function in a CT equipped environment, the needle position can be monitored (as described in the medical protocol) and corrections can be applied during the procedure. The robot controller enables, for the lowest motion speed, a 0.02° for each axis, therefore enabling accurate repositioning of the needle when necessary.

Large orientation workspace. The pre-planning phase (see protocol in Section 2.2) imposes the definition of safe needle trajectories from an external point (on the patient’s skin) to the target point inside the tumor. As the trajectory is linear, the robot must be capable of orienting the needle guiding device with wide angles in order to ensure safe insertion trajectories.

Real-time tissue resistance monitoring. It was experimentally determined and demonstrated in [43,44] that the needle will deviate from its trajectory when insertion forces exceed certain values. Furthermore, supported also by researchers from the European Oncology Institute [45], to achieve a linear resistance force from the tissues, a small incision (1 mm) must be made where the needle should penetrate the skin.

Haptic control. While most procedure stages are performed automatically (based on predefined trajectories), both on-site corrections and treatment of new (previously unidentified) tumors are often needed; in such cases, the procedure also requires manual adjustments which should be carried out using a device that enables the doctor to feel, in real time, the tissue resistance.

Maintenance program. Even when working with a robotic device in perfect state, it has been shown that the required accuracy can be reached only with additional monitoring, which clearly emphasizes the need for a strict program of preventive maintenance for all the equipment involved in the procedure, including the CT-scan device.

Sterilizable needle insertion device. As the insertion device comes in close contact with both the needle and the patient, efficient sterilization must be ensured which should not have any effects on the device’s functional parameters.

CT-scan compliance. The entire system must work in the vicinity of a CT scanner, with the robot wrist and needle device inside the gantry. This imposes an electrical protection (shielding) of all the electronics.

Automated safety features. The control system must embed safety features with focus, firstly on the patient, then the medical personnel, the robotic system itself and the environment. As Kuka iiwa is a collaborative robot with multiple native safety features, the team must focus on additional ones related to the medical procedure: automated needle retraction for forces/torques exceeding predefined limits, quick robot removal in case of emergencies and collision management protocol.

Fast manual control. Due to the unique anthropometric characteristics of every patient and the demonstrated error propagation, a complete automated procedure cannot be performed in a real-case scenario. Thus, the robot should also enable efficient, safe and fast manual control during the procedure.

**Figure 5:**
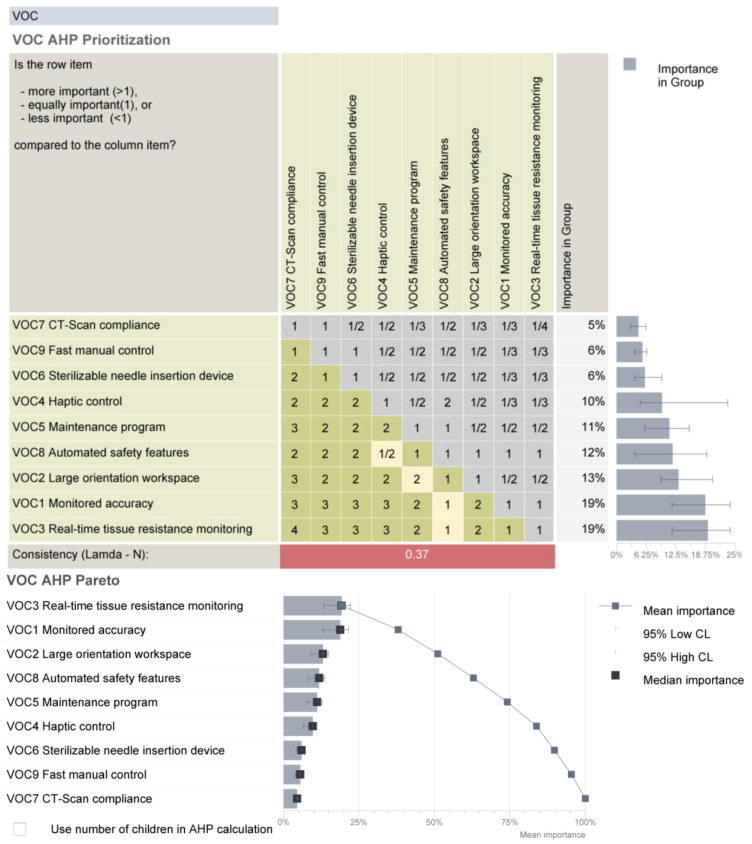
The prioritized requirements for the brachytherapy robotic system.

The requirements were assessed by comparing their relative importance and effort/difficulty to achieve them. The data have been processed using Qualica QFD [46], and the final results are presented in Figure 5.

By definition, all the established requirements must be fulfilled in order to achieve an efficient robotic system, and this analysis highlights that significant research effort must be oriented towards solutions that ensure real-time tissue monitoring and monitored accuracy, which provides the most critical information during the treatment to ensure a safe, reliable, replicable and efficient therapeutic approach.

### 2.4. A Theoretical Study of the Procedure Accuracy

**Figure 6:**
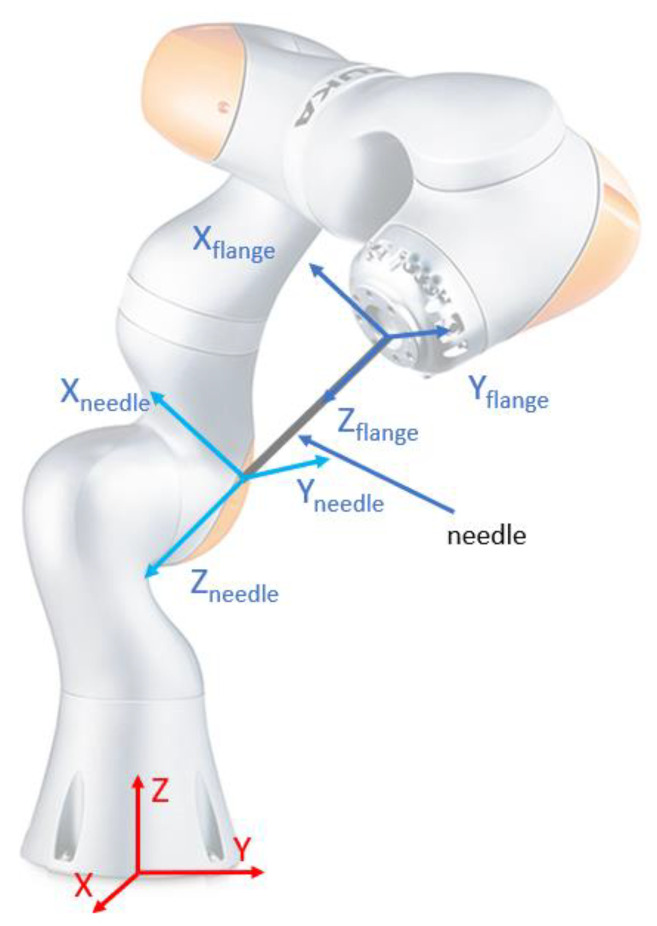
Reference systems for computing needle accuracy.

As shown before, the robotic system used for the needle insertion module’s positioning is the KUKA iiwa LBR R800, which, as described in the technical specifications, provides an overall accuracy of ±0.1 mm. The medical characterization for the radioactive seeds positioning is set to be below 2 mm, which is fulfilled by the previously mentioned robot. Further, a study is proposed in order to assess the dispersion of the final needle tip locations, when errors are introduced at the level of the robot flange in the specified range [47]. As the relative robot–patient position imposes that the brachytherapy instrument will always be located above the patient, the following equation relates the relative displacement between the robot flange and the needle tip (see Figure 6):

(1) $$\left\{ \begin{array}{l} X_{needle}=X_{flange}+l_{needle}\cdot \sin\left(\theta \right)\cdot \cos(\psi )\\ Y_{needle}=Y_{flange}+l_{needle}\cdot \sin\left(\theta \right)\cdot \sin(\psi )\\ Z_{needle}=Z_{flange}-l_{needle}\cdot \cos\left(\theta \right) \end{array}\right.$$

where the parameters used in the simulation are:

(2) $$\left\{ \begin{array}{l} X_{flange},Y_{flange},Z_{flange}:\mathrm{variation}\;\mathrm{between}\;\left[-0.1:0.05:0.1\right]\mathrm{mm}\\ \theta ,\psi :\mathrm{variation}\;\mathrm{between}\;\left[-1\text{:}0.4\text{:}1\right]\;\deg\\ l_{needle}:[50,100,150,200]\mathrm{mm} \end{array}\right.$$

**Figure 7:**
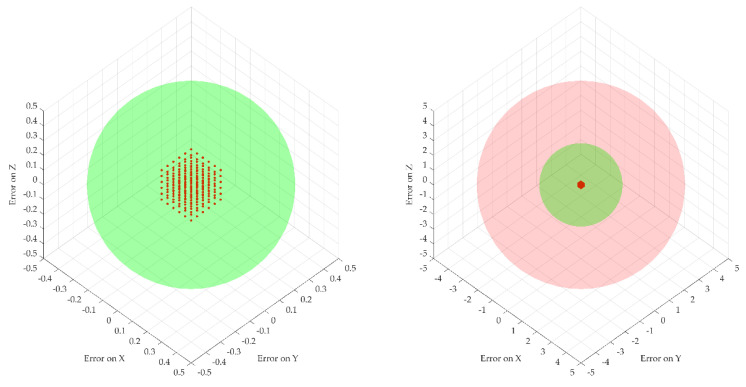
Robot flange positions based on the introduced errors (right, scaled down to [−5 ÷ 5] mm axes scale for comparison with the needle tip data).

The error propagation was studied at four different insertion depths of 50, 100, 150 and 200 mm. Figure 7 illustrates the dispersion of the robot flange locations and the needle tip location for the four different depths. Two spheres were introduced to show the acceptable volume for the needle tip with respect to the ideal coordinates (with a radius of 2 mm—the maximum acceptable positioning error defined by medical data) and the non-acceptable volume (with a radius of 5 mm) to emphasize the actual dispersion of the needle coordinates location.

**Figure 8:**
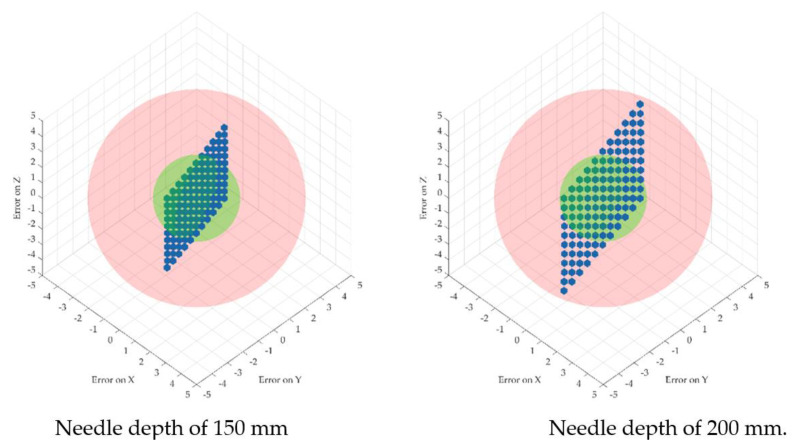

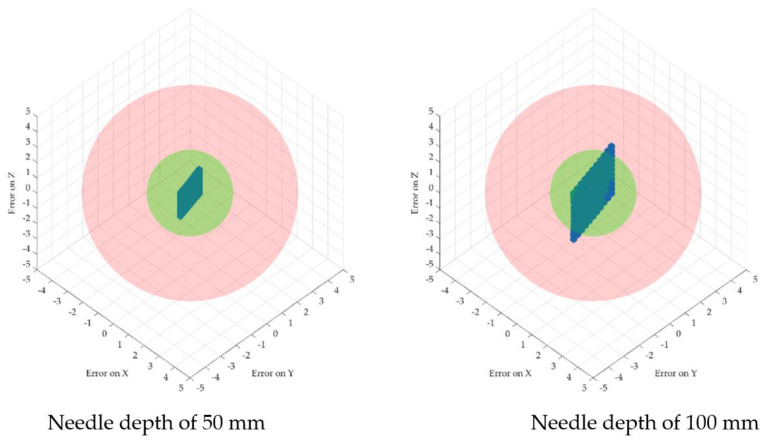
Needle tip-positioning errors for different depths.

Figure 7 and Figure 8 illustrate the fact that for a very small dispersion error at the level of the flange, the error for the needle tip exceeds the maximum allowable errors with the increase in the tumor depth. In particular, a random number generator is used to replicate the actual coordinates at the robot flange, adding an inaccuracy to the “theoretical” coordinates. This is represented by the point cloud illustrated in Figure 7, where the red dots represent the real possible locations of the robot flange with respect to a single “theoretical” set of coordinates. The real needle tip location is determined with respect to the point cloud created earlier when the insertion is completed at various depths (50, 100, 150 and 200 mm). While the dispersion of the real flange locations is enclosed in a rectangle with sides of 0.2 mm, the resulting error at the needle tip (illustrated graphically in Figure 6) is growing exponentially, reaching up to 5 mm at the depth of 200 mm.

This issue illustrates the criticality of Step 13 in the medical protocol which imposes the validation of the needle orientation angles (using the CT data) before inserting the needle into the tumor.

### 2.5. Experimental Analysis of the Needle Insertion and Retraction Forces

This section of this paper describes the experimental setup for determining the insertion forces at the tip of the needle using different scenarios. For each scenario, 5 insertions were performed using different target points. The insertions were made in ex vivo tissue (fresh porcine liver).

- Scenario no.1: five insertions with the needle perpendicular to the targeted area (vertical) using 250 mm/s speed and 10 mm spacing between the insertions, 60 mm deep.
- Scenario no.2: five insertions with the needle perpendicular to the targeted area (vertical) using 125 mm/s speed and 10 mm spacing between the insertions, 60 mm deep.
- Scenario no.3: five insertions with the needle perpendicular to the targeted area (vertical) using 25 mm/s speed and 10 mm spacing between the insertions, 60 mm deep.
- Scenario no.4: five insertions with the needle perpendicular to the targeted area (vertical) using 12.5 mm/s speed and 10 mm spacing between the insertions, 60 mm deep.
- Scenario no.5: five insertions with the needle perpendicular to the targeted area (vertical) using 250 mm/s speed and 150 degrees rotation between the insertion point and target point, 10 mm spacing between the insertions, 60 mm deep.
- Scenario no.6: five insertions with the needle perpendicular to the targeted area (vertical) using 125 mm/s speed and 150 degrees rotation between the insertion point and target point, 10 mm spacing between the insertions, 60 mm deep.
- Scenario no.7: five insertions with the needle perpendicular to the targeted area (vertical) using 12.5 mm/s speed and 150 degrees rotation between the insertion point and target point, 10 mm spacing between the insertions, 60 mm deep.

**Figure 9:**
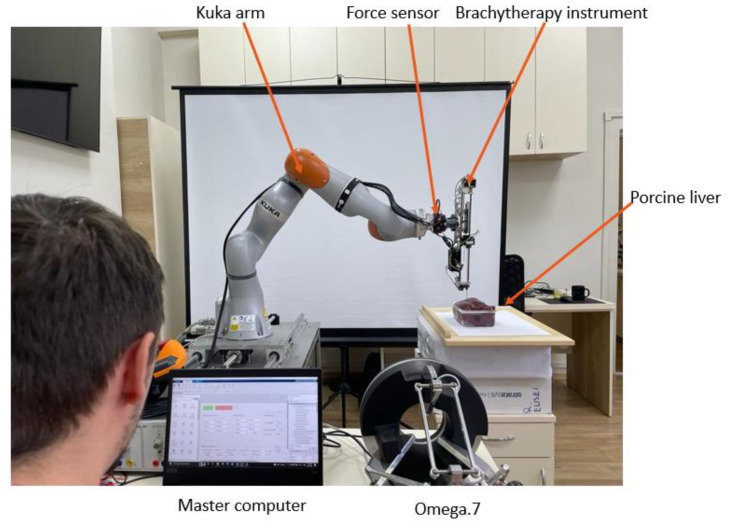
The experimental setup for force measurements.

The experimental setup is presented in Figure 9. The forces are recorded with the FT 300 sensors using a sample rate of 200 readings/min.

## 3. Results

### 3.1. Force Analysis

**Figure 10:**
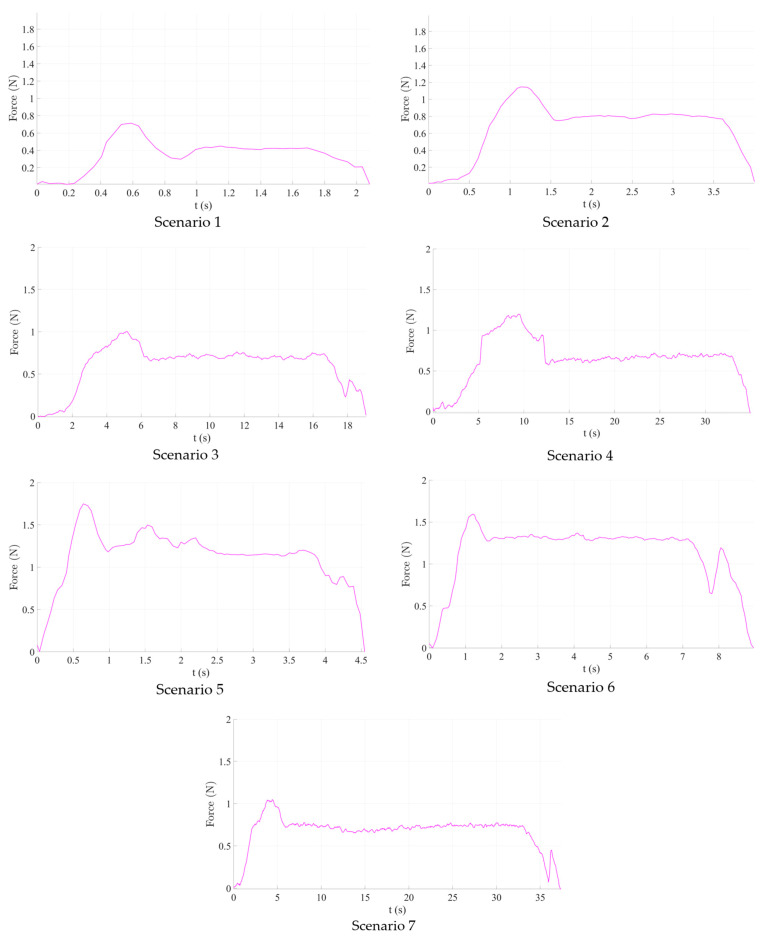
Graphical representation of the experimental results.

The results obtained during the experimental tests are graphically represented in Figure 10.

Scenario no.1. The maximum registered insertion force reached was 0.715 N during tissue penetration, while the mean value was 0.412 N. Immediately after penetration, the registered insertion force decreased to approximately 0.3 N before finally stabilizing. Scenario 2 (125 mm/s insertion speed) recorded a higher insertion force (approximately 1.2 N), similar to Scenario 4 (12.5 mm/s insertion speed). Nevertheless, the mean value of the measured insertion force had increased, nearly doubling (0.6–0.8 N). As the insertion speed decreased, more samples were obtained, which may be considered noise because the F/T sensor baud rate was constant throughout all experiments.

As a result of the first set of studies, it is obvious that for a linear insertion of the needle in the hepatic parenchyma, a higher speed will ensure a much smoother insertion, thus a better overall accuracy of the medical procedure.

Scenarios 5–7, which combine needle insertion and needle rotation at various speeds show, perhaps surprisingly, higher both maximum (between 1 and 1.8 N, depending on the insertion speed) and mean insertion forces, which means that, at least for the rotation speed used in this experiment (50–16.7–4.5 deg/s) it did not help to decrease the insertion force. This leads to the conclusion that the needle rotation would be useless for a needle insertion instrument, using the Fransen needles (Argon Medical Devices (USA), Cluj-Napoca, Romania). Even more, at least for Scenario 5 (250 mm/s insertion speed), the insertion force has fluctuated quite a lot within all insertion tests, proving an unwanted instability, which will not be helpful in a pre-programmed force control algorithm for the insertion of the needle.

As a result of the second set of experiments, the use of a supplementary rotation motion for the needle is detrimental for the medical procedure, increasing the overall resistance force of the tissue. However, the test was conducted directly on the liver parenchyma, and further experimental tests are planned in the future, to test whether in some insertion instances (skin, fatty superficial tissues), this rotation motion could improve the overall procedure efficiency.

The forces obtained during the experimental analysis are equivalent to kinetic friction forces and will be further used to compute the normal forces and ultimately determine the wear of the mechanism.

### 3.2. Normal Forces Decomposition

Starting from the insertion and retraction forces recorded during the experimental tests, using Equation (3), we can calculate the normal forces exercised while inserting and retracting the needle.

(3) $$F=\frac{Fr}{\mu }$$

where *Fr* represents the friction, *μ* represents the friction coefficient and *F* is the normal force.

**Figure 11:**
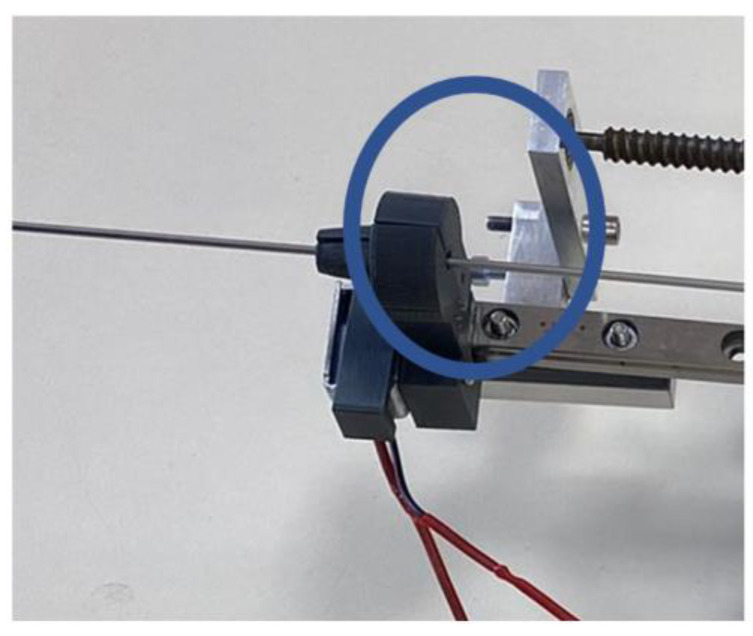
Needle centering device.

First element that is in direct contact with the needle is the centering device (Figure 11).

The centering device has been 3D printed using acrylonitrile butadiene styrene (ABS), with a conical shape, composed of two symmetrical parts. One is fixed, while the other is actuated by a solenoid, ensuring physical contact between the needle (made entirely of stainless steel, surgical grade) and the jaws of the centering device, thus acting as a guide for the needle. Two types of friction forces are generated during the needle placement:

- Sliding friction, which is generated during the needle insertion between the centering devices’ jaws and the needle and during the stylet extraction, generated between the canula and the stylet;
- Static friction, generated during the stylet extraction between the centering devices’ jaws and the canula (which is kept in place while the stylet is removed).

The friction coefficient in dry conditions is: μ = 0.2 for the ABS–stainless steel combination and μ = 0.5 for the stainless steel–stainless steel combination [48].

**Figure 12:**
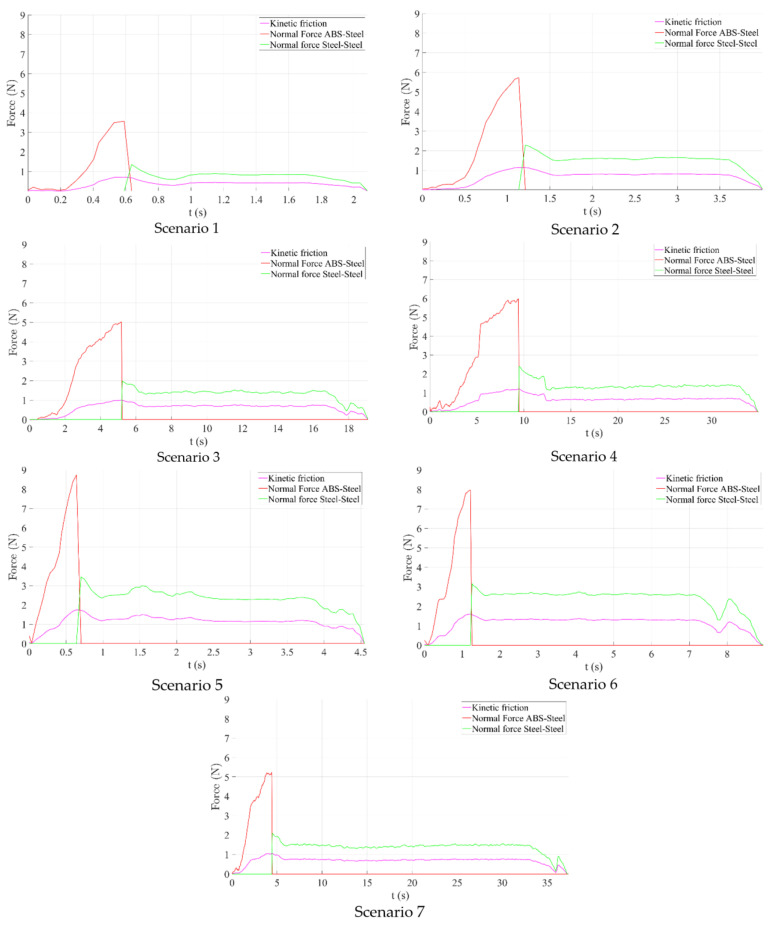
Normal force computation.

Figure 12 presents the time history diagram of the normal forces determined after analyzing the friction between the needle and the centering device and between the stylet and the canula for all seven scenarios previously defined. The maximum values of the normal forces are presented in Table 4:

The purple line in Figure 12 represents the measured force during insertion and retraction, the red line represents the normal force between the jaws of the centering device (ABS) and the cannula of the needle (steel) and the green line represents the normal force between the cannula (steel) and the stylet (stylet). The lowest recorded normal force has been obtained in the first scenario (for the 250 mm/s insertion speed), both between the centering device and the needle and between the stylet and the cannula. The grasping force exerted by the jaws of the centering device must be at least equal (or greater) to the sliding friction force between the stylet and the cannula. Friction forces recorded in the case when the needle was also rotated during insertion were considerably higher but further investigations are required due to the fact that only sliding friction was computed excluding the revolving friction.

### 3.3. Wear Analysis

Next step in analyzing the lifecycle of the medical instrument for brachytherapy is to determine the wear for the main components. When analyzing the relative motion between two contact surfaces, wear can be defined as the loss of material due to the mechanical action between the parts (caused by friction forces) [49]. The previously established normal forces are used for wear ascertainment associated with the jaws of the centering device. Archard equation [50] is used in order to calculate the wear.

(4) $$W=k\cdot \frac{P\cdot L}{H}$$

where *W* is the wear in cubic millimeters, *k* is the Archard wear coefficient (non-dimensional), *P* is the normal force (Newtons), *L* is the sliding distance (millimeters) and *H* is the hardness of the wearing object (MPascal). The first-order derivative of Equation (4) represents the wear rate in Equation (5), where “*v*” represents sliding velocity in mm/s.

(5) $$\dot{W}=k\cdot \frac{P\cdot v}{H}$$

In this case, the wear rate represents the loss of material for the centering device and the needle, respectively, between the needle stylet and the cannula when a motion is performed on the entire length of the needle at various velocities.

Table 5 presents results for computing the wear and the wear rate for all seven scenarios, where the wearing part is composed of the ABS jaws of the centering device. The maximum sliding length is the total length of the needle: 200 mm. The wear coefficient for ABS–steel friction is 10^−4^ and for the steel–steel friction is 0.007 [51,52]. A value of 20.5 HB was used for ABS, while 235 HB was used for steel. As can be observed, the highest wear and wear rates were obtained in the case of Scenario 5 and the lowest in the case of Scenarios 1 and 7.

## 4. Discussion

When discussing the lifecycle of a mechanical device, there are several aspects that need to be taken into consideration: the materials of the components, the working environment/conditions, the wear during operation of the mechanism, etc. The importance of above aspects becomes more apparent when the device in question is a medical device used to provide care or treatment to patients in need.

This paper presents a series of analyses, both theoretical and experimental, to determine certain behaviors of the components of a robotic system used for brachytherapy with the scope of improving the safety of the patient, of the surgeon and of the mechanism during the brachytherapy procedure. After describing the robotic system and the robotic brachytherapy procedure’s medical protocol, a critical analysis of the robotic system’s technical requirements is presented, the technical characteristics which were analyzed are: monitored accuracy, large orientation workspace, real-time tissue resistance monitoring, haptic control, maintenance program, sterilizable needle insertion device, CT-scan compliance, automated safety control and fast manual control. The critical analysis pointed out that during the medical procedure real-time tissue monitoring and monitored accuracy are of highest importance. During the needle insertion, due to its flexibility, the needle would deviate from its initial path as a consequence of high forces and as such real time tissue resistance monitoring is important for this aspect in order to minimize needle deflection during insertion. By monitoring the accuracy of the procedure, the effectiveness of the treatment is validated during the procedure.

In order to thoroughly minimize the positioning error of the needle during the medical procedure, a theoretical study was conducted regarding the dispersion of the final needle tip location by introducing errors at the robot flange’s level. Due to the length of the needle, this study revealed that for a very small dispersion error at the flange’s level, the needle’s error increases and exceeds the allowable limit proportionally with the insertion depth. The statistics revealed by this study could possibly improve the overall procedure accuracy by overcompensating for the error during the planning phase.

The next step in defining the lifecycle of the medical device was to perform a tribological analysis of sliding elements in order to determine the friction forces and the wear of the components. The forces present during the brachytherapy procedure were experimentally determined for the tribological analysis. The experimental setup consisted of the robotic system and ex vivo porcine liver. Using the robotic system several needle insertions were performed using different scenarios. The forces were recorded during the experiment using a force sensor of 0.3 N resolution. The lowest insertion force was recorded while inserting the with the highest possible speed, but further investigations are required in order to establish the accuracy of the insertion at high speed vs. low speed.

The insertion and retraction forces were considered as friction forces and were further used to compute the normal forces during the insertion. The normal forces were computed using previously determined friction coefficients and were further used to determine the wear of the contact elements during the needle insertions. Two types of interactions were found: between the needle made of stainless steel and the centering device made of ABS plastic and between the stylet and the cannula. Wear and the wear rate were investigated for both cases revealing that the minimum wear is obtained when the needle is inserted with highest speed and the maximum wear was recorded while inserting the needle at highest speed while simultaneously rotating the needle. The standard deviation during the rotation motion was determined to be 0.001368 mm^3^ and the mean value was 0.006291057 mm^3^, meaning that at one full passing of the needle through the centering device, the jaws of the device will lose 0.006291057 mm^3^ of its volume; by setting a safety coefficient of 1.5 mm^3^, the jaws of the centering device may be used for approximately full 238 insertions.

Regarding the contact between the stylet and the cannula, by keeping the same safety coefficient, this study reveals that the needle is capable of achieving approximately 105 insertions. However, after interrogating a few medical professionals, it was identified that the lifecycle of a sterilizable needle is significantly shorter than that predicted by the wear of the stylet inside of the canula due to frequent bending of the needles.

## 5. Conclusions

This paper presented a study design and experimental setup of a robotic system for brachytherapy using tribology analysis. The robotic system is composed of a serial collaborative Kuka iiwa 7 R800 robotic arm and a multi-needle instrument, capable of inserting up to six needles in one brachytherapy session, which are controlled using a unified control system embedding an Omega 7 haptic device with force-feedback. The accuracy of the procedure was assessed utilizing a variety of scenarios for inserting and extracting needles from porcine ex vivo tissue, as well as the forces and friction that go along with them. This study also provided and critically reviewed the technical parameters of the system components. The friction forces were computed, in order to calculate the normal forces and the wear during insertion, as well as centering the device jaw’s and needle’s lifetime.

## Figures and Tables

**Table 1:** Cancer statistical data with reference to 5-year survival rates and incidence.

Cancer Location	5-Year Survival Rates (%) Related to Detection Time				Incidence (Number)	
	Overall	Local	Regional	Distant	New Cases	Deaths
Breast	90	99	86	29	290,560	43,780
Colon and rectum	65	91	72	15	151,030	52,580
Esophagus	20	46	26	5	20,640	16,410
Kidney	76	93	71	14	79,000	13,920
Liver	20	35	12	3	41,260	30,520
Lung and bronchus	22	60	33	6	236,740	130,180
Skin melanoma	93	99	68	30	99,780	7650
Pancreas	11	42	14	3	62,120	49,830
Prostate	98	>99	>99	31	268,490	34,500
Stomach	32	70	32	6	26,380	11,090

**Table 2:** Clinical cases which could benefit from brachytherapy.

No.	Diagnosis	Therapy	Evolution
**Tumors locate in the prostate**			
1	Prostate adenocarcinoma T3NoMo, G8,iPSA = 18 ng/mL (2013)	Hormone therapy (HT) + External radiotherapy (RT) 76 Gy	Local recurrence. A complement of brachytherapy seeds in 2013 could have avoided the recurrence.
2	Prostate adenocarcinoma T2NoMo, G6, iPSA = 11 ng/mL (2011). Right seminal bladder recurrence	Permanent implant of iodine 125	Local recurrence. A complement of brachytherapy seeds would avoid a mutilating surgery that the patient refused.
3	Prostate adenocarcinoma T2aNoMo, G6, iPSA = 9 (2012). Prior rectum cancer surgically removed	Hormone therapy (HT)	Death. Robotic brachytherapy would have saved the patient.
4	Bladder cancer, T2NoMo	Radical cystectomy	Partial cystectomy and robotic brachytherapy would have avoided the mutilating surgery with a better quality of life.
**Tumours located in the liver**			
1	Rectosigmoid cancer stage IV (liver and pulmonary metastases), Radio- and Chemotherapy (RCT), surgery	Palliative chemotherapy. Radiofrequency ablation	Focal brachytherapy would have performed better on the liver metastases.
2	Oesophagus cancer, stage III, RCT. Local recurrence and liver spread. Cirrhosis	Supportive care	Death. Brachytherapy (oesophagus and liver) would have extended the patient survival with good life quality.
3	Unifocal hepatocellular carcinoma over cirrhosis. Inoperable due to comorbidities	Sorafenib	Death. Local brachytherapy would have extended the patient survival.
Tumours located at the rectum level			
1	Rectal cancer, RCT, surgery. Local recurrence	Palliative chemotherapy	Local and distant recurrence. Brachytherapy would have improved the prognosis avoiding (or delaying) metastases.
2	Rectum (stage III) and prostate Synchronous Adenocarcinoma T3NoMo	RCT + surgery	Prostate brachytherapy would have prevented the surgery and all its complications (incontinence, urinary infection).
3	Epidermoid carcinoma anal canal. T4N2Mo, RCT. Local recurrence. Surgery (rectum amputation)	Surgery	Initial brachytherapy would have avoided the recurrence and thus the amputation.
4	Inferior rectum adenocarcinoma T2NoMo	Rectum amputation	Local excision and brachytherapy would have avoided the second, mutilating, surgery.
**Thoracic tumours (lungs and breast)**			
1	Pulmonary metastases following testicle cancer, multiple recurrences	Chemotherapy, surgeries (testicle, ganglions, lungs) External pulmonary radiotherapy	Death. Brachytherapy would have avoided the recurrences.
2	Epidermoid pulmonary cancer. Local inoperable recurrence	Chemotherapy, palliative radiotherapy	Death. Brachytherapy would have, at least, extended the survival.
3	Thoracic sarcoma	Surgery, radiotherapy	Brachytherapy would have provided the same outcome with much lower toxicity.
4	Retroperitoneal sarcoma, positive margins resection, irradiated	Surgery, RT, CHT	Local recurrence. Brachytherapy could have complemented the external dose of radiation to avoid recurrences, without the increase in intestinal toxicity.

**Table 3:** The medical protocol for robotic assisted brachytherapy.

Step	(1)—Preplanning
1.	The patient undergoes a complete non-invasive imagistic investigation (CT—compulsory and MRI—if needed) for the exact definition of the tumor(s) location.
2.	The CT images are analyzed and the following parameters are defined: a The radiation dosage and type. b The number of needles. c The matrix distribution of the needles. d The linear trajectories which have to avoid the proximity of high-risk areas (organs penetration, blood vessels, nerves) and high-density tissues (cartilages, bones).
3.	Using CT-scan equipment which has an external laser-based fixed coordinate system, the patient position for the procedure is established to enable: a Comfortable fixed patient position during the entire procedure (as only local anesthesia will be used); b Easy access to all the predefined trajectories for the robot with the needle insertion module positioned above the patient.
4.	A set of markers is positioned on the patient to enable: a An accurate reproduction of the patient position with respect to the external laser-based coordinate system; b A clear view of the needle insertion points achieved through: i. Markers highly visible under the CT-scan; ii. A needle guiding plate where the holes of interest are marked.
5.	A second tomography is performed, using the same CT-scan, to validate the positions of all the markers (if needed fine adjustments are made for the final positions).
6.	The relative robot–patient position is optimized to ensure that all the targeted points can be reached (are located within the robot workspace) and the final trajectories are validated.
	**(2)—CT-guided robotic brachytherapy**
7.	Using the numbered mounting holes the robot is positioned on the mobile CT-scan couch followed by the patient, whose position will be reproduced using the existing markers (step 4). The patient and robot are calibrated with respect to the laser coordinate system and the transformation matrix for the transformation of the patient coordinates into the robot coordinates is introduced.
8.	The robot performs the homing and then positions the needle insertion module in a predefined point, above the patient, but not very far from the area of interest.
9.	The coordinates of the pairs of points (Insertion—Target) are loaded using a secured USB drive to avoid any human errors.
10.	The first needle is positioned from the current (neutral point) into the Insertion point oriented on the linear trajectory defined by the first pair of points. Due to the external markers the doctors will be able to see any misalignments before any invasive action has been performed.
11.	The needle is introduced into the patient at a depth of 40–50 mm followed by a quick scan aimed to validate the trajectory.
12.	If needed, small trajectory corrections are applied.
13.	In case the trajectory has an error higher than 0.2°–0.5° (depending on the target depth) the needle is retracted and introduced again.
14.	The needle is introduced until the targeted point and then released from the insertion module.
	For each following needle the steps 10–14 are repeated until all the needles are in place.
15.	The robot is retracted into the home position to allow full access to the inserted needles.
16.	The needles are connected to dedicated equipment that will deliver the radioactive seeds based on the treatment protocol.
17.	After the treatment the needles are extracted from the patient body and the patient is removed from the CT-scan mobile couch.
18.	The robot is removed, and the procedure is completed.
	**(3)—Patient follow-up**
19.	The patient evolution is monitored and based on his/her evolution subsequent treatments are scheduled.
20.	All the information is stored in a database recording the robot parameters, procedural times as well as all the medically relevant data such as needle accuracy and treatment efficiency.

**Table 4:** Normal forces acting on the needle maximum values.

Scenario	Maximum Normal Force	
	Between Needle and Centering Device [N]	Between Stylet and Canula [N]
1 (250 mm/s)	3.57	1.36
2 (125 mm/s)	5.73	2.28
3 (25 mm/s)	5.02	1.99
4 (12.5 mm/s)	5.99	2.392
5 (250 mm/s + rotation)	8.74	3.45
6 (125 mm/s + rotation)	7.97	3.16
7 (12.5 mm/s + rotation)	5.24	2.08

**Table 5:** Wear computation.

Scenario	Wear [mm^3^]		Wear Rate [mm^3^/s]	
	ABS–Steel	Steel–Steel	ABS–Steel	Steel–Steel
1 (250 mm/s)	0.003482927	0.008102128	0.004353659	0.01012766
2 (125 mm/s)	0.005590244	0.013582979	0.003493902	0.008489362
3 (25 mm/s)	0.004897561	0.011855319	0.000612195	0.001481915
4 (12.5 mm/s)	0.005843902	0.014250213	0.000365244	0.000890638
5 (250 mm/s + rotation)	0.008526829	0.020553191	0.010658537	0.025691489
6 (125 mm/s + rotation)	0.00777561	0.018825532	0.004859756	0.011765957
7 (12.5 mm/s + rotation)	0.005112195	0.012391489	0.000319512	0.000774468
Min value	0.003482927	0.008102128	0.000319512	0.000774468
Max value	0.008526829	0.020553191	0.010658537	0.025691489

## Data Availability

The data presented in this study are available on request from the corresponding author.
